# New Mode of Improving Grass Lands

**Published:** 1811-01

**Authors:** 

**Affiliations:** Norfolk


### *New Mode of improving Grass Lands. By Mr.* SALTER *of Norfolk.*

(Communications to the Board of Agriculture.)

As Mr. Coke, President to the Norfolk Agricultural Society, has expressed himself in terms of approbation so highly gratifying to me upon my method of improving poor pastures and boggy meadows, and particularly when he honoured me with a visit this summer for the purpose of examining a meadow, which was then in its highest state of improvement with a crop growing upon it ; I readily comply with your request, and have sent you the best information I can of the method, which I have pursued now ten years in that line of farming ; a method, which originated in accident, but which has ever since been carried on systematically.

At Michaelmas 1795, I entered upon this farm, consisting of upwards of six hundred acres, of which the greatest part is wet, springy, cold land. There were at that time, about one hundred acres of bad meadow, so over-run with rushes, sedges, and all sorts of aquatic plants, that no sheep had ever been known to be pastured upon them ; whereas, for the last eight years, I have not had a single instance of a rotten sheep. I first cut the rivulet, which runs through the meadows, three hundred and fifty-two rods in length, (reckoning seven yards to a rod,) and eight feet wide. I also cut one thousand one hundred and sixteen rods of open drains ; and the turf or sods, which came out of them, I laid to dry in the months of February and March ; and as soon as dried I gathered them on large heaps of sixty and a hundred loads, and burnt them to ashes. On the 2d of April, 1796, I dibbled about two acres of that part of the meadow which was most dry, and immediately I carried on, in half-load tumbrils with broad wheels, about fifteen loads *per* acre of the turf ashes : then I sowed sixteen or eighteen pounds of Dutch clover, and four bushels of ray grass ; *i. e.* eight or nine pounds of Dutch clover, and two bushels of ray grass *per* acre. These I brushed with a pair of harrows bushed, and rolled three or four times with a very heavy roll, in order to make them as firm as possible. Upon that part of the meadow which was boggy and rushy, I laid from eighty to an hundred tumbril loads *per* acre of sand, fine gravel, and mould, as I could most conveniently come at them, cutting and carrying away every hillock of waste earth which I could find. Having harrowed and rolled this, I dibbled upon every acre two bushels of summer vetches, one bushel of early grey peas, peas, and two bushels of Poland oats, all mixed together; and then sowed Dutch clover and ray grass as I did upon the dry part of the meadow, in which I omitted the oats, knowing they would not have succeeded.

In dibbling thus, the holes ought to be four inches square from each other, and from two to four seeds should be put into a hole. Peas and vetches thus growing upon grass land, whether on low meadows or dry uplands, have never failed with me of having excellent effect. They entirely destroy moss, and ameliorate the soil. It is to be observed, that upon dry uplands I omit the ray grass. When the crop is forward in the pod, I mow it for hay; and as soon as it is dry, I put it upon small cocks, and then on to large ones, so as to prevent the leaves from falling off. If I do not want this hay for my sheep, I cut it for my horses; and it is so nutritious, that it serves both as hay and corn.

In the year 1803 I grew thirty acres of vetches, peas, and oats, managed as above: the crop was not less than from two and a half to three loads *per* acre: by a load, you know, I mean as much as a waggon drawn by four horses can carry. In the same year I sowed one hundred acres of turnips three times over, and at last lost my whole crop, except a single turnip. I had 532 breeding ewes to maintain in the following winter. Having provided thirty troughs twelve feet long, and a straw-cutting machine, (which with a horse will cut ten coombs an hour), I cut the hay made of the vetches, peas, and oats, and thus fed my sheep, which produced me a greater number of lambs, and a greater quantity of milk for the lambs, than I ever had from turnips. They were kept in the straw-yard from the 10th of October to the middle of April; and thus I kept them last year, and shall always keep them whilst I remain upon a heavy-land farm. They eat the straw well, and make a far better yard of muck, than that from bullocks. It was by much the best muck I ever had, except a yard of muck, where I fatted 220 pigs, by scattering peas about the yard.—As a proof of my success in lambs in the year 1804, my shepherd, Thomas Nunn, gained one of the premiums (five guineas) from the Society.

In February, 1805, a seventeen-acre meadow was become solid; I filled up the open drains, and cut nine hundred and ninety-five rods of under-drains, which I filled up part with bushes and part with stones. Upon April 4th, I began to dibble vetches and peas; then carried on about 15 loads *per* acre of out-hollowing, muck, and mould together; sowed Dutch clover and ray grass, and brushed them as usual: the crop was abundant—I got 63 loads of hay from the 17 acres. This was the meadow which Mr. Coke, Mr. Gurdon, you and other other gentlemen admired so much, and deemed an improvement in the management of grass land deserving of high commendation.

I will not omit to tell you from what accident this method of managing grass lands originated :—In the year 1784 I had a piece of grass land eaten up by grubs : I sowed vetches upon it, and harrowed them. They produced so good a crop, that I have continued the practice to the present time. Indeed I carry it on in every instance where I can ; for whenever my lays of clover fail, instead of breaking them up, I dibble or drill vetches early in the spring. It is worthy of remark, that the ray grass and Dutch clover upon my meadows come much earlier, and grow faster, than the lays upon my arable land. Be assured, that I shall have pleasure in attending you to Reepham to examine your pasture there, in order to *salter* them, as you are pleased to call my method of improving grass land.

P. S. I as often use the drill roll for the vetches and peas, as I do the dibbles : please to observe also that I never plough, pare, or scarify my grass lands.

